# POINeT: protein interactome with sub-network analysis and hub prioritization

**DOI:** 10.1186/1471-2105-10-114

**Published:** 2009-04-21

**Authors:** Sheng-An Lee, Chen-Hsiung Chan, Tzu-Chi Chen, Chia-Ying Yang, Kuo-Chuan Huang, Chi-Hung Tsai, Jin-Mei Lai, Feng-Sheng Wang, Cheng-Yan Kao, Chi-Ying F Huang

**Affiliations:** 1 Institute of Clinical Medicine, National Yang-Ming University, Taipei, Taiwan, ROC; 2 Institute of Biotechnology in Medicine, National Yang-Ming University, Taipei, Taiwan, ROC; 3 Institute of Bio-Pharmaceutical Sciences, National Yang-Ming University, Taipei, Taiwan, ROC; 4 Institute of BioMedical Informatics, National Yang-Ming University, Taipei, Taiwan, ROC; 5 Department of Computer Science and Information Engineering, National Taiwan University, Taipei, Taiwan, ROC; 6 Armed Forces Peitou Hospital, Taipei, Taiwan, ROC; 7 Institute for Information Industry, Taipei, Taiwan, ROC; 8 Department of Life Science, Fu-Jen Catholic University, Taipei County, Taiwan, ROC; 9 Department of Chemical Engineering, National Chung Cheng University, Chia-Yi County, Taiwan, ROC

## Abstract

**Background:**

Protein-protein interactions (PPIs) are critical to every aspect of biological processes. Expansion of all PPIs from a set of given queries often results in a complex PPI network lacking spatiotemporal consideration. Moreover, the reliability of available PPI resources, which consist of low- and high-throughput data, for network construction remains a significant challenge. Even though a number of software tools are available to facilitate PPI network analysis, an integrated tool is crucial to alleviate the burden on querying across multiple web servers and software tools.

**Results:**

We have constructed an integrated web service, POINeT, to simplify the process of PPI searching, analysis, and visualization. POINeT merges PPI and tissue-specific expression data from multiple resources. The tissue-specific PPIs and the numbers of research papers supporting the PPIs can be filtered with user-adjustable threshold values and are dynamically updated in the viewer. The network constructed in POINeT can be readily analyzed with, for example, the built-in centrality calculation module and an integrated network viewer. Nodes in global networks can also be ranked and filtered using various network analysis formulas, i.e., centralities. To prioritize the sub-network, we developed a ranking filtered method (S3) to uncover potential novel mediators in the midbody network. Several examples are provided to illustrate the functionality of POINeT. The network constructed from four schizophrenia risk markers suggests that EXOC4 might be a novel marker for this disease. Finally, a liver-specific PPI network has been filtered with adult and fetal liver expression profiles.

**Conclusion:**

The functionalities provided by POINeT are highly improved compared to previous version of POINT. POINeT enables the identification and ranking of potential novel genes involved in a sub-network. Combining with tissue-specific gene expression profiles, PPIs specific to selected tissues can be revealed. The straightforward interface of POINeT makes PPI search and analysis just a few clicks away. The modular design permits further functional enhancement without hampering the simplicity. POINeT is available at .

## Background

Protein-protein interactions (PPIs) are critical for virtually every biological process. Diverse experimental techniques for detecting PPIs have been developed and have improved dramatically in the last decade, i.e., yeast two hybrid (Y2H), affinity chromatography, co-immunoprecipitation (Co-IP), and fluorescence resonance energy transfer (FRET) [1,2]. Advances in chip techniques also enabled the applicability of protein chips in detecting PPIs under diverse conditions in a high-throughput manner [1]. High-throughput screenings of PPI have also been carried out for various organisms, including yeast [2], worm [3], fruit fly [4], and human [5]. The large amount of data accumulated from various sources has posed a grand challenge in data reliability and the searching, analysis and filtering for PPI.

In order to facilitate PPI searching, a number of systems provide batch input and output functionality, such as Genes2Networks [6], Ulysses [7], T1DBase [8], and the Arabidopsis Interactions Viewer [9]. Genes2Networks provides a dynamic linkable three-color web-based network map, with a statistical analysis report that identifies significant intermediate nodes used to connect the query lists. In Ulysses, users can project model organism gene properties onto homologous human genes to perform interolog analysis. T1DBase provides various aspects of information regarding type 1 diabetes and includes an interaction network viewer. In addition to the type 1 diabetes PPI network, this viewer can also be used to construct other networks of interest. The Arabidopsis Interactions Viewer mainly focuses on the Arabidopsis PPI information and is designed for an interactome of Arabidopsis predicted from interacting orthologs in yeast, worm, fruit fly, and human. Using these services and packages, networks in different species or conditions can be searched, downloaded and visualized.

The above described services can easily perform searches and construct networks from user-supplied queries. However, the analyses of these networks require other software packages, which may have incompatible input formats and complex interfaces. There are several network analysis tools for PPI network evaluation, such as Pajek [10], CentiBiN [11], and NetworkAnalyzer [12]. These tools support the calculation of node centralities, such as degree centrality, closeness centrality, betweenness centrality, and cluster coefficient, to name a few. The analysis of node centrality characteristics in a network serves as an efficient means to understand the relative roles and features of each node. Various studies have suggested that proteins with larger numbers of interactions (hubs) are more critical [13-16], although the interpretations of this phenomenon differ [17,18]. Missing/losing these hub proteins is likely to result in death or developmental defects in the organisms. Using the topological features in biological networks, nodes playing different roles can be ranked and selected.

These web services and software tools are valuable to the processing of PPI networks. However, one has to comprehend several systems/tools to fully exploit the knowledge hidden in the biological networks. Therefore, an integrated web service is provided in this study for searching, analyzing, and observing a PPI network. The PPIs can also be filtered with expression profiles of various tissues and NCI60 cell lines. Integrated systems with a simplified workflow for handling PPI networks will facilitate the utilization of PPI networks. In this manuscript, we discuss three case studies on the "putative risk gene identification", "hub prioritization for the midbody interactome", and "filtering PPI with tissue-specific expression profiles". Researchers can use POINeT to address various questions by combining PPI networks, tissue expression profiles and sub-network analysis functions in one website. We have previously established an ortholog-based protein interactome database, POINT [19,20], by using the concept of interologs [21,22], whereby conserved PPIs in various species can be mapped to human PPIs. Here, we have extended the PPI search function in POINT to a new and updated PPI network web service, POINeT . A comparison between the functions of POINeT and POINT is listed in Table 1.

**Table 1:** Comparison between POINT and POINeT features.

**Features**	**POINT**	**POINeT**
Human interologs	Yes	Yes
Experimental PPI	No	Yes
Experimental PPIs from other species	No	Yes
Interologs prediction for other species	No	Yes
Network Construction	No	Yes
Network Viewer	No	Yes
Network Topology Analysis	No	Yes
Hub Prioritization and Ranking	No	Yes
Tissue-specific expression profile filtering	No	Yes
Network export and download	No	Yes

The improvements of POINeT over POINT are listed and compared.

## Implementation

**Figure 1:**
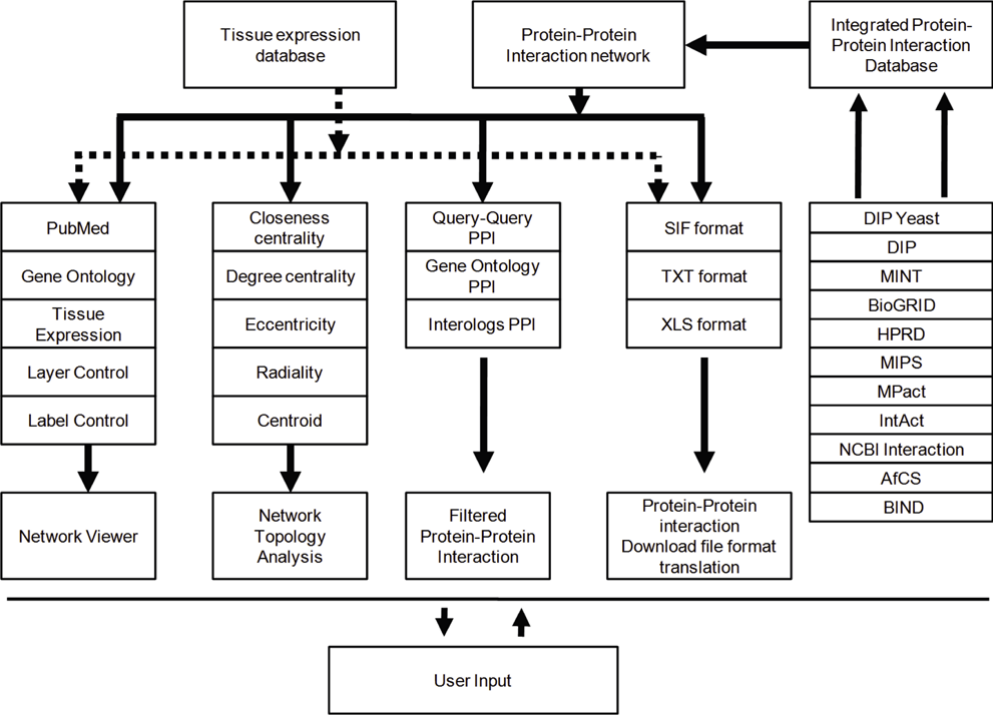
**The overall system architecture of POINeT**. POINeT is able to provide efficient PPI network related services in one query through the integration of data from various sources.

We have adopted several network analysis measurements from the literature [23], such as closeness, degree, eccentricity, radiality and centroid centralities, and implemented several tools to automatically prioritize PPIs and nodes in a biological network in POINeT. Figure 1 illustrates the overall system architecture of POINeT. These tools are described in the following sections.

### Protein-protein interaction Data Resources

PPIs included in POINeT were merged from various sources, including DIP [24], MINT [25], BIND [26], HPRD [27], MIPS [28], CYGD [29], BioGRID [30] and NCBI interaction . Since different PPI databases use different ID systems, these disparate IDs have been mapped to the NCBI Gene IDs. Therefore, PPIs with different designations from various sources may map to the same interactions. The components of POINeT are combined systematically to meet the needs of the users. For each PPI, additional information was provided, including PubMed IDs, links to the literature reporting this PPI, and Gene Ontology (GO) annotations [31]. Users may input a set of proteins using their corresponding Gene Symbols, Gene IDs or UniProt accession numbers to query the PPI data. Table 2 lists the numbers of PPIs collected from various data sources by POINeT. Interologs can be incorporated into the query result to enrich the potential PPIs in the output networks.

**Table 2:** Protein-protein interaction data sources incorporated in POINeT.

**Data Source**	**Version or Download Date**	**Number of Interactions Included**	**References**	**References Details**
BioGRID	2.0.37	202,244	Stark *et al*. (2006)	Stark, C., Breitkreutz, B.J., Reguly, T., et al. (2006) BioGRID: a general repository for interaction datasets, *Nucleic Acids Res*, **34**, D535–539.

IntACT	2008/2/11	121,560	Hermjakob *et al*. (2004)	Hermjakob, H., Montecchi-Palazzi, L., Lewington, C., et al. (2004) IntAct: an open source molecular interaction database, *Nucleic Acids Res*, **32**, D452–455.

HPRD	version 7	37,107	Peri, S. et al. (2003)	Peri, S. et al. (2003) Development of human protein reference database as an initial platform for approaching systems biology in humans. Genome Research. 13:2363–2371.

MPact	2005/12/22	12,955	Ulrich G. et al. (2006)	Güldener U, Münsterkötter M, Oesterheld M, Pagel P, Ruepp A, Mewes HW, Stümpflen V(2006). MPact: the MIPS protein interaction resource on yeast. Nucl. Acids Res. 2006 34: D436–D441

DIP	2008/1/14	50,048	Xenarios *et al*. (2000)	Xenarios, I., Rice, D.W., Salwinski, L., et al. (2000) DIP: the database of interacting proteins, *Nucleic Acids Res*, **28**, 289–291.

MINT	4.0	99,773	Zanzoni *et al*. (2002)	Zanzoni, A., Montecchi-Palazzi, L., Quondam, M., et al. (2002) MINT: a Molecular INTeraction database, *FEBS Lett*, **513**, 135–140.

CYGD	2007/1/25	33,984	Guldener *et al*. (2005)	Guldener, U., Munsterkotter, M., Kastenmuller, G., et al. (2005) CYGD: the Comprehensive Yeast Genome Database, *Nucleic Acids Res*, **33**, D364–368.

BIND	2006/5/25	41,603	Bader *et al*. (2003)	Bader, G.D., Betel, D. and Hogue, C.W. (2003) BIND: the Biomolecular Interaction Network Database, *Nucleic Acids Res*, **31**, 248–250.

MIPS	2007/1/1	1,363	Mewes *et al*. (2004)	Mewes, H.W., Amid, C., Arnold, R., et al. (2004) MIPS: analysis and annotation of proteins from whole genomes, *Nucleic Acids Res*, **32**, D41–44.

The numbers of PPIs from various data sources as collected by POINeT. The total number of PPIs is not the sum of all PPIs from various sources since there are numerous redundant entries.

### Protein-protein interaction Query Flow

**Figure 2:**
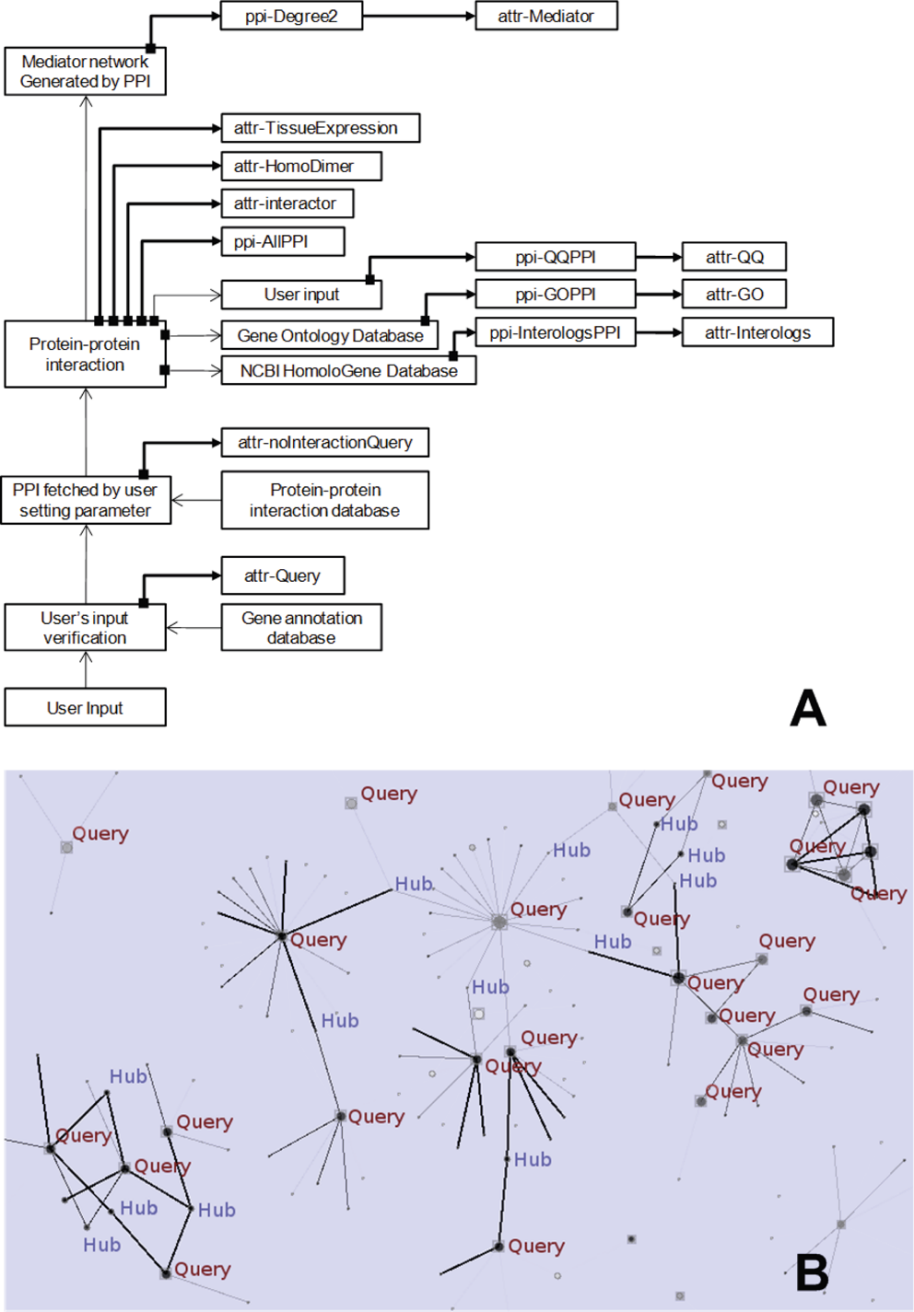
**The analysis results and downloadable items provided by POINeT**. In downloadable items, (A) attr-Query has the record of the input query of genes. The table ppi-AllPPI contains all the PPIs resulting from the query. The nodes involved in ppi-AllPPI will be identified and recorded in the attr-Interactor table. The nodes with degree >= 2 are defined as mediators and recorded in the attr-Hub table. The nodes of the attr-Hub table form a network, which is denoted as ppi-Degree2. If two interactors of one interaction were both present in the attr-Query table, this interaction will be documented in ppi-QQPPI. Interactors in the ppi-QQPPI network will be recorded in the attr-QQ table. POINeT will merge ppi-QQPPI, ppi-GOPPI, and ppi-InterologsPPI into the ppi-FilteredPPI. This network contains PPIs with higher reliabilities and certain biological significances. (B) A simple PPI network is provided to illustrate the components of the network. Query nodes are marked with red circles; mediators (nodes connecting more than two nodes) other than query nodes are marked with blue circles. QQPPI are shown in black lines. GOPPI are shown in red lines. InterologousPPI are shown in green lines.

The workflow for querying, filtering and downloading PPIs is depicted in Figure 2A. Briefly, the user inputs the query terms (genes or proteins), which will be recorded as attr-Query, into POINeT to search for all available PPIs, referred to as ppi-AllPPI. If a query has no available PPI, POINeT stores it as attr-noInteractionQuery. If certain filtering criteria are set in the query page, such as 'Number of iterations' or 'Number of literature references', the number of PPIs included in ppi-AllPPI will change accordingly. Subsequently, the nodes involved in ppi-AllPPI will be in the attr-Interactor table and the degrees of these nodes will be calculated. Since the proteins outside of the query protein set could serve as a mediator in PPI network, such as a regulator or an adapter protein, nodes with a degree >= 2 are defined as mediator and recorded in the attr-Mediator table. The mediators are nodes (query and/or non-query) connecting any two query proteins. This will form another network, which removes all nodes with a degree = 1 and is denoted as ppi-Degree2. This network can reduce the complexity of network visualization and illustrate how queries are connected through these mediators. These mediators may be an important member of the sub-network around the query proteins. If a query node interacts with itself and forms a homodimer, this node will be recorded in the attr-HomoDimer table. Furthermore, if two interactors of one interaction were both present in the attr-Query table, this interaction will be documented in ppi-QQPPI. Interactors in the ppi-QQPPI network will be recorded in the attr-QQ table. Figure 2B illustrates various components in a PPI network. Interologs in different species can be inferred systematically using the NCBI HomoloGene database. These interologs' PPI will be recorded in the ppi-InterologsPPI table. Using the gene2go mapping table provided by NCBI, whether two interactors of one PPI share the same GO annotation will be noted, resulting in the ppi-GOPPI network. Finally, if interactors of ppi-QQPPI are present in the attr-Hub table, these interactors will be placed in the attr-QH table, which denotes that a node exhibits both a query and a hub in the network. POINeT will merge ppi-QQPPI, ppi-GOPPI, and ppi-InterologsPPI into ppi-FilteredPPI. This network contains PPIs with relatively reliable and certain biological significances. This network, which is smaller than ppi-AllPPI, can be visualized and analyzed with ease and extended with other selected features. These described tables can be downloaded in multiple formats.

### Protein-protein interaction filtering component

#### Interaction Filtering Using Biological Characteristics

POINeT provides three types of PPIs, including PPIs among queries (Query-Query PPI), PPI in which interactors share the same GO terms (GO PPI), and interologs' PPI. Moreover, various literature references, i.e. [32], have shown that proteins sharing the same GO terms are more likely to interact with each other. POINeT has the option to match PPIs sharing the same GO terms. Using the ortholog information available for various species, PPI networks can be mapped to different model organisms. For every species available in POINeT, the interolog PPIs can be inferred from the experimental PPIs in other species. For example, predicted human PPIs can be inferred from the experimental PPIs of mouse, worm, fly, yeast, and even *Arabidopsis *(though the number of predicted PPIs from the latter is much smaller than those of the other model organisms). In short, POINeT provides functions to filter experimental PPIs and to infer interolog PPIs. Through these different settings, PPIs among proteins with similar biological functions can be filtered and revealed, permitting an in depth analysis of unsorted PPIs.

#### Interaction Filtering Using Tissue-Specific Expression Profiles

SymAtlas [33] has included tissue-specific expressions of 79 tissue types from human and mouse. The expression profiles of NCI60 cell lines from SymAtlas are also incorporated. SymAtlas used human and mouse U133A microarray from Affymetrix, along with custom-made chips, GNF1H (for human) and GNF1M (for mouse). Each probe on the microarray can be mapped to corresponding genes with conversion tables provided by Affymetrix and the Genome Institute of the Norvatis Research Foundation (GNF). With the information available, the expression levels of interactors (genes) in PPI networks can be presented in an integrative way based on user-selected tissues or cell lines. In addition to tissue-specific genes, tissue-specific PPIs can also be filtered and inferred with these expression profiles.

### Protein filtering component

#### Protein Filtering Using Centralities

The analysis of node centrality characteristics in a network serves as an efficient means to understand the relative roles and features of each node. Several centrality measurements are available in POINeT, including degree centrality, closeness centrality, eccentricity, radiality, and centroid values. The meanings and detailed description of these centralities is available in textbook [34]. Degree centrality is the number of edges associated with a node, normalized to a quantity from 0 to 1 by dividing by the maximum associated edge number in the sub-network. High-degree nodes in a protein interaction network tend to correspond to proteins that are essential and may be a good predictor of their biological importance [13]. Closeness centrality (CC) can identify nodes closer to other nodes in the biological network [35]. In our implementation, larger values indicate that the paths between the given nodes to all other nodes are shorter. Eccentricity is the longest distance required for a given node to reach the entire network. In graph theory, the set of vertices with the minimum eccentricity is denoted as the center of a graph. Radiality centrality (RC) is similar to closeness centrality. The path lengths from one node to all other nodes are subtracted by the maximum shortest path length of the network, then summed and averaged, and the absolute value taken [36]. Compared to nodes with smaller radiality, nodes with larger values are closer to all other nodes. Centroid values identify optimal positions (nodes with positive values) in a network. Before the calculation of centrality values, POINeT will identify sub-networks included in the ppi-AllPPI. An individual sub-network can be selected for centrality analysis. Some centralities by definition can only be evaluated on connected graphs, such as CC, RC, and Centroid. The results of these calculations can all be downloaded directly from the web page. These centrality values can also be applied to prioritize nodes in the network.

#### Protein Filtering Using Sub-Network Specificity Scores

Biological networks are likely comprised of several sub-networks or functional modules contributing to various diverse biological processes [37]. A node may have negligible impact on the global network or global properties, yet is influential on a sub-network with specific functionality. Therefore, it is desirable to devise a measurement to reflect the sub-network specificity of nodes. Moreover, it has been shown that data fusion using rank combinations can improve the specificity of the ranking results [38].

Thus, two scores were proposed and merged in this work. One score is the ratio between the sub-network degree and the global degree of a given node:



where *i *is the designated node,  is the degree of node *i *in sub-network *N*, and  is the degree of node *i *in the global network. The  score refers to the proportion of interactions contributed to the sub-network by node *i*. A larger score implies that the node has higher preference over the given sub-network.

The other score is based on the statistics of node degree distributions in randomly sampled sub-networks. A bootstrap method has been used to sample the degree of node *i *in 1000 random sub-networks with the same size as the designated one. The Z-score for the degree of node *i *is calculated as follows:



where *μ *is the mean of the node *i *degree distribution in random sub-networks, and *σ *is the standard deviation of the random degree distribution. The Z-score provides a statistical evaluation on the significance of the degree of node *i*, namely whether the degree of node *i *is likely to have resulted from the random sampling of sub-networks.

These two scores are highly correlated since they are based on the same concept – the differential distribution of node degrees in sub-networks and the global network. If most of the interactions of a node are contributed to a given sub-network, we assume that this node is significant to this sub-network and not to the other sub-networks or the global network. That is, the node is "specific" to the designated sub-network. However, there are minor disagreements on the local ranks given by these two scores. To make the most out of the two scores, a data fusion model has been applied to merge the two scores [38]:



where  is the rank of node *i *by the  score, and *R*(*z*_*i*_) is the rank of node *i *by the *z *score. S3 refers to the "Sub-network Specificity Score," which is the rank with the combination of the two proposed scores.

### Output component

The query results of POINeT can be downloaded in multiple formats, including Excel, sif (simple interaction format), and txt formats. Using the exported sif format, ppi-AllPPI, ppi-Degree2, ppi-FilteredPPI and all attributes can be downloaded. Tissue-specific expression profiles can also be exported into individual attribute files. The query results in sif format can be easily integrated with tissue-specific expression profiles, and visualized in CytoScape [39]. Also, plain text files can be downloaded as well. However, Excel and txt formats do not support the export of tissue-specific expression profiles.

### Network viewer

POINeT provides a straightforward viewer with sufficient functionalities. No additional software installations are required. Networks and tissue-specific expression profiles can be visualized directly in the browser. The viewer supports zooming and panning of the networks. The concept of layers in geographic information system (GIS) [40] was adopted. Different output results were defined as different layers. Through the selection of different layers, ppi-QQPPI, ppi-GOPPI and ppi-InterologsPPI can be displayed individually or as a merged network, ppi-FilteredPPI. The labels on each layer can also be turned on/off, as can the labeling of selected nodes. Finally, nodes can be selected to display the associated interactions, PubMed IDs, and Gene Ontology annotations, and provide the links to external databases. Also, tissue-specific expression values are treated as attributes of the nodes in the network. Using the concept of layers adopted from GIS, different tissue expressions can be selected and displayed for the same nodes to facilitate the analysis and comparison of these expression profiles. The network viewer provided by POINeT permits users to observe gene expression levels of the same PPI network in different tissue types.

## Discussion

### Putative Risk Gene Identification

PPI network analysis is an emerging field for the identification of, for example, disease related genes. By analyzing the gene expression and combining with the integration of omic data sets from different species to construct the PPI network, Migual et al., identified potential genes associated with higher risk of breast cancer [41]. A genes network of disorders linked by known disorder-gene associations led to discover a single graph-theoretic framework in disease gene associations, indicating the common genetic origin of many diseases [42]. These reports suggest that one application of biological networks is the identification of novel marker genes for diseases and the study of interactions among these marker genes. In the case of schizophrenia, a population-based analysis has revealed four genes, DAAO (DAO), DAOA, DTNBP1 and NRG1 [43], to be associated with the schizophrenia. Certainly it would be interesting to discover any associations among these genes in terms of biological networks and their potential involvement in specific biological pathways.

**Figure 3:**
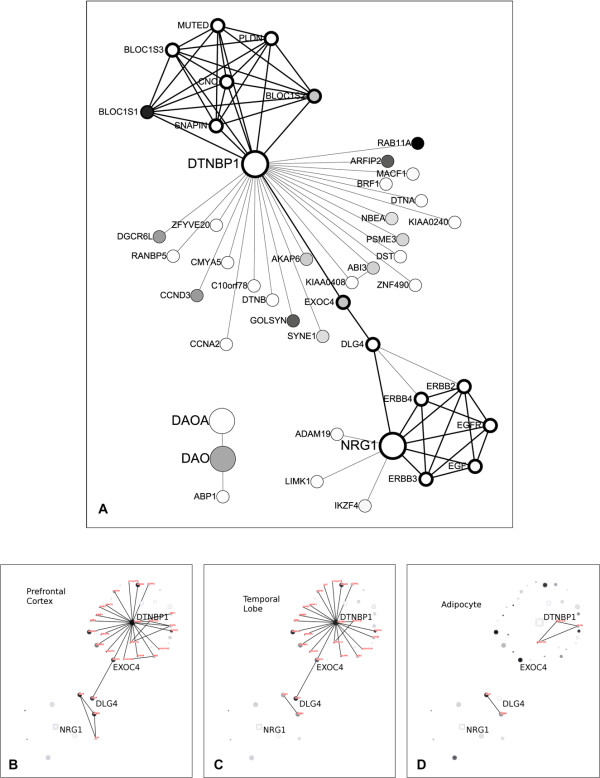
**Connections between the schizophrenia risk genes DTNBP1 and NRG1**. (A) DLG4 and EXOC4 are positioned on the path between DTNBP1 and NRG1. Without DLG4 and EXOC4, the links between DTNBP1 and NRG1 would be broken. The gene expression patterns of the nodes in the temporal lobe are labeled with differential levels of grey, where darker shades denote higher expression levels. This figure is generated using CytoScape. The same network in two brain tissues, (B) Prefrontal Cortex and (C) Temporal Lobe, reveal the presences of interactions among DTNBP1, DLG4 and EXOC4. (D) Whereas in adipocyte (which is not related to brain and schizophrenia), most of the interactions are missing.

Using these four genes as queries, there are interesting links between DTNBP1 and NRG1 (Figure 3A). DTNBP1 and NRG1 are both involved in fully connected cliques. Two nodes lie between DTNBP1 and NRG1; these are DLG4 and EXOC4. The interactions among DTNBP1, DLG4 and EXOC4 are present in various brain tissues, such as prefrontal cortex and temporal lobe, which are known to be related to schizophrenia etiology (Figure 3B and 3C). Most of the interactions in this sub-network are missing in other un-related tissues, such as adipocyte (Figure 3D). DLG4 is known to be involved in nicotine dependence [44]. There is no known association between DLG4 and schizophrenia in the literature; notwithstanding this, because there are constant controversial debates on the genetic factors contributing to schizophrenia, DLG4 is greatly deserving of further investigation. Similarly, EXOC4 is known to be involved in the exocyst complex, which is critical for the release of neurotransmitters [45]; at present its functional involvement in schizophrenia is unknown. The roles of DLG4 and EXOC4 in schizophrenia remain to be explored, and the two genes might serve as putative risk markers with potential for further studies.

### Hub Prioritization for the Midbody Interactome

Recently, various proteomes focusing on specific spatiotemporal conditions have been elucidated, such as the midbody [46]. For these proteome results, it would be interesting to devise the interactions among the protein components, further extending the proteome into the interactome. The midbody is an important organelle formed in the later stage of cytokinesis, and is required for the separation of two daughter cells after cell division. The midbody interactome [46] has been listed as an example on the POINeT website. Based on a literature review, we have extended this set to 190 midbody-related proteins. The first question we asked was whether the limited numbers of midbody proteins, identified in the recent proteomic screen, participate individually in the process of cytokinesis or whether groups of the midbody proteins interact with each other and form a network. The second was how to fill in the missing gaps in the constructed midbody PPI network and identify novel targets participating in the process of cytokinesis. Using POINeT can answer, at least in part, these two questions, i.e. identify the PPI network of the midbody. Besides PPI and network analysis, ranking/prioritization of nodes in networks may also contribute to the identification of novel components of the midbody proteome.

**Figure 4:**
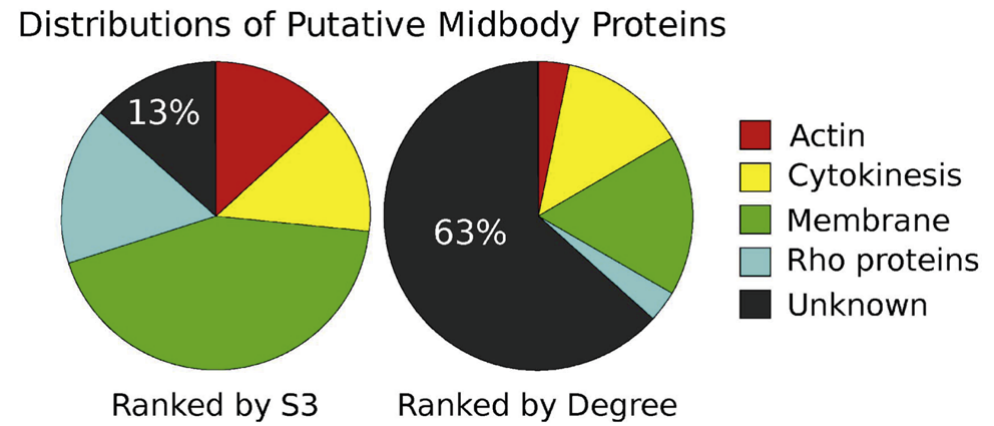
**Distributions of putative midbody proteins in the top 30 mediators ranked by the sub-network specificity score (S3) and the degree centrality**. Four types of proteins are considered as putative midbody proteins, including actin-related, cytokinesis-related, membrane associated, and rho proteins. Other proteins with unrelated annotations were classified as unknown. As compared to degree centrality, S3 can enrich the proportion of putative midbody proteins into the top-ranked mediators. This implies that the ranking given by S3 could be used to refine the composition of the midbody proteome.

The mediators in the midbody interactome have been ranked using two measurements: the hub degree and the sub-network specificity score (S3). In order to evaluate these two scores, the top 30 proteins ranked by these two scores were listed and analyzed for their ability to enrich midbody-related proteins (Table 3). Figure 4 illustrates the results. Four types of proteins were considered to be putative midbody proteins [46], including actin-related, cytokinesis-related, membrane-associated, and Rho proteins. The top 30 mediators ranked by S3 contain only 13% unknown proteins, with 87% putative midbody proteins, whereas the top 30 mediators ranked by degree centrality contain 63% unknown proteins, with only 37% putative midbody proteins. Figure 4 illustrates that the sub-network specificity score can effectively enrich the proportion of proteins highly related to the designated sub-network. The involvements of the S3 top-ranked genes in the midbody proteome have also been, at least in part, confirmed experimentally (manuscript in preparation). These results suggest that S3 could be employed to refine the midbody proteome, identify novel midbody proteins and rank these proteins, which may be complement to proteomics studies.

**Table 3:** Top30 mediators with prioritized sorting excluding midbody queries.

Ranks by Hub Degree	Gene Symbol	Hub Degree	Total Degree	Annotation	Ranks by S3 Score	Gene Symbol	Hub Degree	Total Degree	Annotation
1	GRB2	36	407		1	HTR3A	2	2	membrane
2	YWHAZ	27	391		2	KIAA0133	2	2	membrane
3	IKBKE	24	328		3	PPP1R14B	2	2	
4	TRAF6	24	369		4	DOCK7	2	3	Rho
5	HLA-B	20	273	membrane	5	GEFT	2	3	Rho
6	MAP3K3	18	173		6	LCT	2	3	membrane
7	ACTB	17	187		7	OPHN1	2	3	Rho
8	YWHAG	16	309		8	PLEKHG2	2	3	Rho
9	RIPK3	15	88		9	MYT1	2	3	
10	IKBKG	15	155	cytokinesis	10	PLEKHM2	2	3	membrane
11	MCC	14	217		11	TOR1AIP1	2	3	membrane
12	EGFR	14	261	membrane	12	MALL	2	3	membrane
13	MYC	13	322		13	ARPC5	5	9	actin
14	TP53	12	315	cytokinesis	14	FLOT2	3	6	membrane
15	CASP3	11	139		15	ESPL1	2	4	cytokinesis
16	EIF1B	11	153		16	ASPM	2	4	cytokinesis
17	CDH1	10	80	membrane	17	CASC3	2	4	membrane
18	PRKCA*	10	181	Rho	18	CD163	2	4	membrane
19	VHL	10	208	membrane	19	MCF2L	2	4	Rho
20	SRC	10	217	cytokinesis	20	DIS3L2	2	4	
21	ACTA1	9	103	actin	21	KTN1	3	7	cytokinesis
22	DISC1	9	113		22	ARPC4	5	13	actin
23	EPB41	9	128		23	SRGAP1	2	5	Rho
24	TNFRSF1A	9	128		24	RPRM	2	5	membrane
25	CFTR	9	135	membrane	25	SEC24D	2	5	
26	PRKAB1	9	153		26	NRAP	2	5	actin
27	FYN	9	161	cytokinesis	27	PLP1	2	5	membrane
28	SMAD3	9	193		28	SEPT11	2	5	cytokinesis
29	GH1	8	76		29	ABCC2	2	5	membrane
30	EIF6	8	101		30	ACP6	3	9	membrane


Putative Midbody Related Proteins			11	(37%)	Putative Midbody Related Proteins			26	(87%)
	Actin		1	(3%)		Actin		3	(10%)
	Cytokinesis		4	(13%)		Cytokinesis		4	(13%)
	Membrane		5	(17%)		Membrane		13	(43%)
	Rho proteins		1	(3%)		Rho proteins		6	(20%)
Unknown Proteins			19	(63%)	Unknown Proteins			4	(13%)

The top 30 proteins ranked by hub degree (left) and sub-network specificity score (S^3^, right) and analyzed for their ability to enrich midbody-related proteins.

* PRKCA is both a Rho protein and a cytokinesis related protein. It is classified as Rho protein to simplify the ratio calculation.

### Filtering PPI with Tissue-Specific Expression Profiles

**Figure 5:**
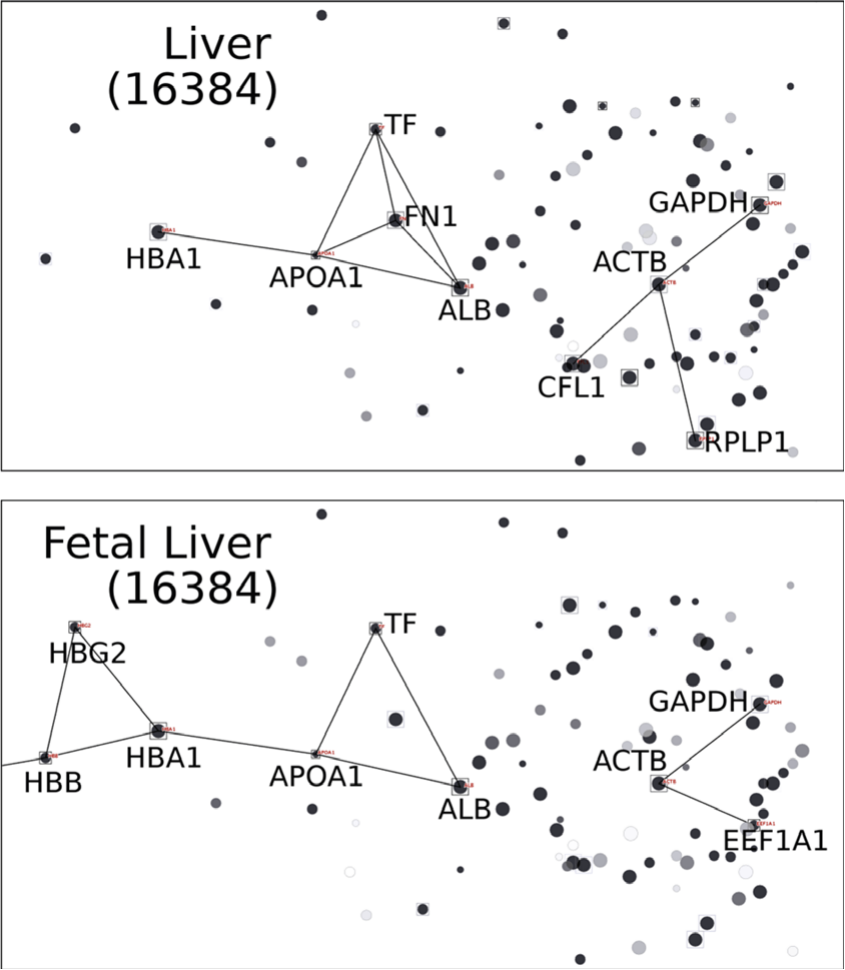
**PPI network of liver filtered by different tissue expression profiles**. The expression levels of the nodes are represented by differential levels of grey. Query nodes are marked with squares. PPIs are filtered with prespecified gene expression level (16384). The PPI networks filtered by liver and fetal liver expression profiles are similar, but some subtle differences can be noted. For example, interactions between HBA1 and HBG2 are present in the fetal liver but not in the (adult) liver. This reflects the actual compositional differences between fetal and adult hemoglobins.

To demonstrate the PPI filtering capability of POINeT, a PPI network specific to liver has been constructed from 173 highly abundant proteins in mass proteomic data of liver [47,48]. Two gene expression profiles from the SymAtlas database were selected: liver and fetal liver. Figure 5 illustrates the results of the tissue-specific gene expression profile filtering. PPIs are shown in the figures when the gene expression levels of the two interacting proteins exceed the specified thresholds.

The threshold selection is dependent on the questions to be addressed. The networks filtered with liver and fetal liver gene expression profiles are largely similar, since two tissues represent the same tissue in different developmental stages. However, some minor differences can be noted. For example, with a higher threshold value of 16,384, it can be noted that interactions between HBA1 (hemoglobin alpha 1) and HBG2 (hemoglobin gamma 2) are less abundant in liver but prominent in fetal liver. It should be noted that this expression threshold is selected to reveal the differences in abundances of genes in adult/fetal livers. Users may set this threshold based on the questions to be addressed. Liver is responsible for the synthesis of hemoglobins in the fetus. In adults, the predominant forms of hemoglobins are composed of 2 beta chains and 2 alpha chains, whereas the fetal hemoglobins are composed of 2 gamma chains and 2 alpha chains. The fetal hemoglobins are replaced by adult hemoglobins after birth. Also, interactions between fibronectin 1 (FN1) and the other three genes, transferrin (TF), albumin (ALB) and apolipoprotein A-I (APOA1), are less abundant in fetal liver. This might be because the expression level of fibronectin 1 in fetal liver is lower than that in adult liver. Up regulation of fibronectin induces hepatic haematopoiesis during the second trimester [49]. Expression level of fibronectin may only become closer to that of the adult liver after that stage. Thus, tissue-specific expression profiles combined with PPI networks are able to capture the subtle differences between different tissues and the interactions therein.

## Conclusion

The modular design of POINeT enables easy extension of the functionalities, including PPI query flow, PPI filtering, and protein filtering component. Limitations of the system exist on less numbers of literature references, incomplete predicting PPI by interolog in empirical study and over explanation of PPI interactions in biological research. However, the processing of PPI networks has allowed several tools to fully exploit the biological networks and integrated systems with PPI networks.

POINeT is intended to be a research tool. The three examples illustrated in the manuscript focus on different applications. The schizophrenia example illustrates how to identify connections between a set of seemly unlinked genes. The interaction between DLG4 and EXOC4 is such a link missing in the original association studies. The midbody example suggests that our S3 measurements may identify new members of a proteome. We have identified 2 proteins as novel members of the midbody proteome by S3 score and confirmed with experiment (data not shown). The fetal/adult liver example illustrates the use of tissue expression profiles in filtering networks. Such application enables the comparison of networks in different tissues.

In short, the information provided by POINeT in terms of PPI network construction and analysis tools is not only capable of shedding light on the intimate interactions of a given dataset, but is also able to prioritize novel mediators and/or markers that may govern various targeted biological processes.

## Availability and requirements

Project name: POINeT

**Project home page: **

**Operating system(s): **Platform independent

**Programming language: **Java, Struts, JSTL, and AJAX

**Other requirements: **POINeT is compatible with most computer systems. It has been tested on Windows (with Firefox, IE6/IE7, Google Chrome), MacOS (with Safari) and Linux (with Firefox). The network viewer should work on any java script enabled browser. However, there may be more browser/OS combinations that have not been tested. Users are welcomed to provide other OS/browser combination, and we will try to make POINeT compatible with these systems.

## Authors' contributions

CYH, CYK, FSW, and JML provided the concept and guidelines for the POINT/POINeT web servers, and modified the manuscript. SAL collected PPI data, implemented the system architecture, and wrote the manuscript. TCC did the experiment for verification of midbody proteins and supported the findings of previous studies. CHC proposed and implemented the ranking scores, and wrote the manuscript. KCH provided the schizophrenia example. CHT explored the applications in disease network analysis. CYY collected and integrated the tissue-specific expression data.
